# Factorial Structure of the Parent-Reported Version of the Inventory of Callous-Unemotional Traits Among Belgian Children: A Theory-Based Model

**DOI:** 10.3389/fpsyg.2022.839785

**Published:** 2022-07-12

**Authors:** Morgane Payot, Christian Monseur, Marie Stievenart

**Affiliations:** Research Unit for a Life-Course Perspective on Health & Education, University of Liège, Liège, Belgium

**Keywords:** Callous-Unemotional, assessment, factorial structure, measurement invariance, confirmatory factor analysis, external validation

## Abstract

The factorial structure of the Inventory of Callous-Unemotional traits (ICU) is still under debate in the current literature and the published models are predominantly based on the empirical results of the statistical analyses rather than on a strong theoretical background. Aimed at overcoming these limits, the current study examined a factorial structure initiated by a theoretical framework for the parent-version of the ICU, based on a community sample of Belgian children aged 3–9 (*N* = 437; *M* age = 5.59; 54.7% boys). Further, the current study investigated measurement invariance across age and gender, and the external validity of this structure. Confirmatory factor analysis (CFA) indicated that empirical factor models from the current literature demonstrated a relatively poor fit to the data. Alternative models were built based on theory, specifically criteria from the DSM-V specifier “with limited prosocial emotions.” CFA supported an 18-item second order model with three first order factors (Lack of conscience, Unconcern about performance, Lack of emotional expression), a second order latent factor (General dimension of CU traits) and a methodological factor encompassing negatively worded items. Results supported measurement invariance across child gender, and to a lesser extent across age. As expected, the general dimension correlated with measures of aggressive behavior, attention problems, internalizing behavior and empathy. The Lack of emotional expression subfactor showed a different pattern of associations in comparison to the two other subfactors. The implications of these findings are discussed, specifically in relation to the DSM-V LPE specifier.

## Introduction

*Callous-Unemotional* (CU) *traits*, corresponding closely to the affective dimension of psychopathy (Hare and Neumann, 2008), appear to be a concept of great interest in the identification of a distinct subgroup among children and adolescents with conduct problems. Indeed, youth with conduct problems and high levels of CU traits show specific cognitive, emotional, biological and social characteristics, which are distinct from youths with only conduct problems (Frick et al., 2014a). This specific subgroup is also at greater risk of developing psychopathy (Burke et al., 2007; Lynam et al., 2007) and severe antisocial outcomes at a later time (Frick et al., 2014b). These differences between youths, with and without CU traits, suggest distinct causal factors for each group (Frick and Viding, 2009; Frick et al., 2014a). As a consequence of the usefulness of CU traits in understanding the heterogeneity of youth with conduct problems, the concept has been included in the fifth edition of the *Diagnostic and Statistical Manual of Mental Disorders* (*DSM-V*; American Psychiatric Association, 2013) in the diagnostic criteria for conduct disorder (CD), as a specifier named “*with limited prosocial emotions*” (LPE). This specifier is defined by four criteria: lack of remorse or guilt; callous/lack of empathy; unconcern about performance at school, at work, or in other important activities; shallow or deficient affects.

Currently, the most widely used tool to assess CU traits in children and adolescents is the *Inventory of Callous-Unemotional traits* (ICU; Frick, 2004). This questionnaire was created to overcome limitations of previous measures, often made up of a limited number of items included in a broader measure of psychopathy and showing limited psychometric properties (Essau et al., 2006; Ray et al., 2016a). Items of the ICU are derived from four items of the Antisocial Process Screening Device (APSD; Frick and Hare, 2001) which loaded consistently on a CU factor in clinical and community samples (Frick et al., 2000). These same four items were also used to form the DSM-V LPE specifier (Kimonis et al., 2015). For the conception of the ICU, additional items were developed from each of these four core items to evaluate a similar content. The final scale includes 24 items with equal numbers of positively or negatively worded items in order to control the effects of response style and item difficulty (Ray et al., 2016a; Waller et al., 2020). Several versions exist based on age, informant (self-reported or other-reported versions) and language.

### Factor Structure of the Inventory of Callous-Unemotional Traits

Many studies have investigated the factor structure of the ICU on different types of samples, age periods and versions. Despite the four factors initially put forward corresponding to the four items drawn from the APSD (Frick and Hare, 2001), the factor structure that was originally the most supported in the literature was a three-factor bifactor model consisting of a general CU factor and three subfactors: *Callous* (lack of empathy, guilt, and remorse for misdeeds), *Uncaring* (lack of caring about others’ feelings and about ones performance in tasks), and *Unemotional* (absence of emotional expression). This structure has been particularly supported with the self-reported version and in adolescent studies, whether in forensic (Kimonis et al., 2008) or community (Essau et al., 2006; Fanti et al., 2009; Roose et al., 2010) samples. However, this factorial structure presents several limitations. Indeed, the Unemotional factor seems to have lower internal consistency than the remaining two factors and generally failed to demonstrate consistent associations with external criteria variables such as conduct problems and aggression (Kimonis et al., 2008; Waller et al., 2015; Cardinale and Marsh, 2020). Therefore, past research questioned the relevance of these items in the assessment of the specificity of the Unemotional dimension linked to CU traits (Waller et al., 2015; Cardinale and Marsh, 2020). Moreover, the fit indices for this factorial structure were often lower than expected (Kimonis et al., 2008; Willoughby et al., 2015; Ray et al., 2016a). Finally, this factorial structure has little support for the other-reported versions and in middle childhood (Roose et al., 2010; Waller et al., 2015). Currently, a model with two correlated factors (Callous and Uncaring) is the most supported structure in the literature, with the number of items varying between authors (Houghton et al., 2013; Hawes et al., 2014; Willoughby et al., 2015; Gao and Zhang, 2016; Yoshida et al., 2019). In particular, the ICU-12 by Hawes et al. (2014) with 12 items and the ICU-11 (Colins et al., 2016), which omits the only remaining item from the Unemotional factor from the ICU-12, are supported in various age periods such as in adults (Wang et al., 2017), adolescents (Zhang et al., 2019; Thøgersen et al., 2020; Allen et al., 2021), middle-aged children (Hawes et al., 2014; Waller et al., 2015; Wang et al., 2020), in various cultures and with the self-reported or the other-reported versions.

Despite the high density of studies evaluating the factor structure of the ICU, only a handful of studies have examined the factorial structure of the ICU in preschool children. However, early childhood could be a particularly relevant period for the understanding of CU traits, given the emergence of the emotions linked to conscience (empathy and guilt) and the high levels of aggressive behavior (Kochanska and Thompson, 1997; Kochanska et al., 2002). Based on these elements, early evaluation of these traits appears to be crucial. In their community sample of children aged 3–4, Ezpeleta et al. (2013) confirmed a three-factor structure (Callous, Uncaring, Unemotional) with the ICU completed by teachers but they could not replicate the bifactor model, with a general dimension of CU traits and three subdimensions. As in previous studies, a lack of association of the subscale Unemotional with expected variables such as conduct problems or aggressive behavior is reported. Finally, two other studies have supported the ICU-12 identified by Hawes et al. (2014) in a sample of high-risk preschoolers with the parent-version (Bansal et al., 2020b) and in a mixed sample of preschoolers with a CU score combining the parent and teacher responses (Kimonis et al., 2016).

The 2-factor structures, like the 3-factor structures, present limitations. First, the Callous and Uncaring factors may be driven by a methodological variance based on item formulation rather than a construct variance (Ray et al., 2016a). Indeed, the Callous factor largely consists of negatively worded items, while the Uncaring factor largely consists of positively worded items. Second, some items that are derived from the same APSD item/LPE specifier criterion loaded on different factors, supporting the presence of variance due to item formulation. For example, item “Does not care who he or she hurts” and “Tries not to hurt others’ feelings” loaded on the Callous and the Uncaring subfactors, respectively (Hawes et al., 2014). Therefore, the factorial structures which are supported the most by the current literature are not based on a clear theoretical background (Ray et al., 2016a; Yoshida et al., 2019) but are guided by statistical analyses. This constitutes an important limitation for the application of these structures in clinical settings, as the factors do not make sense at a theoretical level. Some studies have attempted to remedy these limitations by considering the item-wording and/or by investigating a 4-factor structure based on the initial APSD items/LPE specifier criteria. For example, studies on adolescent offenders (Kimonis et al., 2008) and on a nationally representative sample of children (Bansal et al., 2020a) failed to confirm the 4-factor model but did not take into account the methodological variance. Among the studies that have included a methodological dimension accounting for item-wording and having investigated a 4-factor model, one supported a model with four correlated factors (Kliem et al., 2020), one supported a three-factor bifactor model (Paiva-Salisbury et al., 2017), and one demonstrated support for these two models (Koutsogiorgi et al., 2021). In general, accounting for item-wording improved the fit of all the models in these studies. However, these three studies involved adolescent samples and the self-reported version of the ICU. Therefore, it would be relevant to investigate whether these same results are found with the other-reported versions of the questionnaire and with younger samples.

Two additional considerations seem important when investigating the factorial structure of the ICU. The first one concerns the presence or absence of a general dimension of CU traits in the factorial structure. Indeed, most studies that have examined the factorial structure of the ICU in preschool or school-aged children have not provided support for the inclusion of a general CU dimension, contrary to the three-factor bifactor model found in adolescent samples. Having a general dimension of CU traits would mean that items within the ICU tap a single underlying CU factor, in addition to specific subdimensions (such as Callous, Uncaring and Unemotional in the 3-factor bifactor model). This general dimension seems crucial since it demonstrates the ability of the ICU to diagnose the global phenomenon of CU traits as defined by the LPE specifier, and not only distinct constructs (such as Callous and Uncaring in the 2-factor structures). Moreover, several authors reported that the latent or summed CU scores present the best and most reliable predictive and concurrent validity, in comparison with subfactors, which tend to show inconsistent correlations with external variables (Waller et al., 2015; Ray and Frick, 2020; Thøgersen et al., 2020).

The second consideration lies in the choice of the model. Indeed, CFA offers two alternatives for modeling multifaceted constructs with a general dimension (in this case, a general CU dimension). First, a second-order model represents subfactors as correlated components of a higher-order construct, which accounts for the shared variance between the subfactors (Kimonis et al., 2008; Dunn and McCray, 2020). Second, a bifactor model specifies on the one hand a general factor which represents the overlap across all the items, and on the other hand several independent and uncorrelated subfactors which account for unique variance in their respective set of items, over and above the variance accounted for by the general factor (Chen et al., 2006). In these two types of models, the general dimension represents the common variance between all observed indicators and can be approximatively interpreted in the same way (Zhang et al., 2021). However, in the second-order model, manifest variables (i.e., responses to the items) are only explained by their subfactor and therefore this model does not permit a distinct separation between the uniqueness of the subdimensions and the communalities of the general dimension. On the contrary, in the bifactor model, manifest variables are explained by the general dimension and their subdimension. Second-order models therefore prevent us from distinguishing between what is only associated with the general dimension and what is only associated with the specific dimensions, while bifactor models allow for a distinction between these components (Chen et al., 2012). Even though the bifactor model is usually used in studies about the factorial structure of the ICU, the possibility of a second-order model should be investigated. Indeed, the relevancy of bifactor models has recently been questioned (Watts et al., 2019), namely due to the tendency of these models to overfit data and to produce out-of-range parameter estimates, and also due to the difficulty of interpretating the subfactors (Bonifay et al., 2017).

### Measurement Invariance of the Inventory of Callous-Unemotional Traits Across Age and Gender

Beyond the factorial structure of the ICU, the measurement invariance^1^ throughout the development of the child must be investigated (Willoughby et al., 2015). It may be that the factorial structure of the ICU does not change with age. For example, the three-factor bifactor model has been confirmed in adolescent (Kimonis et al., 2008), school- (Waller et al., 2015) and preschool-aged samples, despite Ezpeleta et al. (2013) being unable to reproduce the general CU factor in preschoolers. The two-factor model has also been found in school- (Hawes et al., 2014) and preschool-aged samples (Bansal et al., 2020b; Kimonis et al., 2016). On the other hand, the factorial structure of the ICU might change with the development of the child. For instance, emotional expression and regulation evolve throughout child development (Stegge and Terwogt, 2007; Gullone et al., 2010; Pollak et al., 2019) and could affect the Unemotional factor. Furthermore, some items of the ICU could be deemed as inappropriate for young children (for example, items about punctuality or about their performance) (Hawes et al., 2014; Kimonis et al., 2016), which could as a result have an impact on the factor structure. Unfortunately, measurement invariance across age is not systematically investigated in studies on the factorial structure of the ICU, and even less between the preschool and the school-aged periods. A few studies found support for partial measurement invariance across age in a sample of 3 and 4-year-old children with a 3-factor model (Ezpeleta et al., 2013), and in a sample of children aged 5–12 with the ICU-12 (Bansal et al., 2020a). Further research is needed. Indeed, a factorial structure generalizable across childhood would be particularly relevant for clinical and research purposes.

Examining the invariance of the ICU across gender also seems relevant given the evidence for differences between boys and girls in terms of CU traits, externalizing and internalizing behaviors (Cardinale and Marsh, 2020). To date, research has largely supported invariance of the ICU across gender (Essau et al., 2006; Houghton et al., 2013; Gao and Zhang, 2016; Bansal et al., 2020a; Allen et al., 2021).

### External Validity of the Inventory of Callous-Unemotional Traits

Regarding the external validity of the ICU, a meta-analytic review (Cardinale and Marsh, 2020) showed convergent validity between ICU total score and externalizing behaviors such as aggressive behavior and hyperactivity (e.g., Colins et al., 2016; Koutsogiorgi et al., 2021; Ueno et al., 2021), psychopathic traits, and reduced empathy (e.g., Kimonis et al., 2016) across versions and a variety of samples. The subfactors Callous and Uncaring presented associations with external variables relatively similar to the ICU total score (Roose et al., 2010; Ray et al., 2016b; Ueno et al., 2021), but Uncaring showed weaker associations (Ezpeleta et al., 2013; Cardinale and Marsh, 2020). On the contrary, the Unemotional subscale showed low to null correlations with externalizing behaviors and empathy, and therefore presented a divergent pattern of associations relative to the other two subfactors (Essau et al., 2006; Kimonis et al., 2008; Ezpeleta et al., 2013; Colins et al., 2016; Cardinale and Marsh, 2020). Regarding internalizing behaviors such as anxiety or depression, results are mixed in the current literature, but the meta-analysis of Cardinale and Marsh (2020) found small associations between internalizing behaviors and the total ICU score, and the three subfactors. However, results investigating the validity of the ICU could change when accounting for the item-wording. Indeed, Koutsogiorgi et al. (2021) found that the magnitude of associations of the general factor increased and the predictive validity of the subscales decreased when adding a methodological dimension to the 3-factor bifactor model. These authors had also investigated a 4-factor model based on the LPE specifier with additional methodological dimensions (positively and negatively keyed items). They found Callousness (i.e., a lack of empathy) to be moderately and positively associated with aggressive behavior, and negatively associated with anxiety. Unremorseful (i.e., a lack of guilt and remorse) and Unconcern about performance factors showed the same pattern of associations with strong positive associations with externalizing behaviors, moderate positive associations with depression and no association with anxiety. Finally, Unemotional (i.e., the shallow affects criterion) was negatively correlated with anxiety, depression and oppositional defiant disorder. The methodological factors significantly and positively correlated with all these variables. These results supported the relevance of considering a methodological dimension accounting for item wording, and the validity of a model based on the LPE specifier criteria. However, this study is based on self-reports of adolescents, so replications in other samples and with other versions are necessary.

To conclude, previous studies on the factorial structure show several limitations. First, there is a lack of consensus regarding the factorial structure in preschool and school-aged samples, with very few studies in the preschool-period despite the relevance of early identification and prevention. Research is also needed to clarify if, and how, the ICU structure changes throughout child development. Second, the models currently supported in the literature often show marginal fit and are based on statistical analyses rather than on a strong theoretical background. This supports the need to continue the investigation of the factorial structure of the ICU in childhood, with particular attention to the theoretical and clinical meaning of the subfactors, the item-wording, the type of model chosen, and the possibility of including a general dimension of CU traits. Indeed, a structure that makes sense at a theoretical level, such as a structure based on the LPE specifier, could lead to a better understanding of the phenomenon, in research and clinical contexts. For example, such a structure might help the clinician to establish a specific profile of the patient based on the scores obtained for the different subfactors, in addition to facilitating the diagnosis and guiding the subsequent intervention. Finally, the French other-reported version of the ICU has, to the best of our knowledge, not yet been validated, unlike the self-reported version (Pihet et al., 2015). However, questionnaires should be re-assessed when translated and applied in other countries and contexts (de Vet et al., 2011).

### Present Study

The present study intends to further investigate the factorial structure, the measurement invariance across age and gender, and the validity of the French parent-version of the ICU in a Belgian community sample of children aged 3–9.

More specifically, the first aim was to investigate the factorial structure of the parent-version of the ICU by comparing different models through confirmatory factor analysis (CFA). We examined the factorial structures the most commonly accepted in the current literature, namely (Model 1) a three-factor bifactor model (Kimonis et al., 2008), due to its wide initial support especially in the literature on adolescents, (Model 2) a three-factor model (Ezpeleta et al., 2013) and (Model 3) a 12-item two-factor model (Bansal et al., 2020b; Hawes et al., 2014; Kimonis et al., 2016), these two latter models being more supported in preschool and school-aged samples. Moreover, we also examined theoretical models, based on a theoretical background and on item contents to form factors. Specifically, we used the criteria of the specifier “with limited prosocial emotions” (LPE) from the DSM-V (American Psychiatric Association, 2013) to form four factors. Indeed, they are equivalent to the 4 items of the APSD (Frick and Hare, 2001) used to create the ICU and it seemed particularly relevant to group the ICU items in four factors based on what they were originally supposed to assess, namely a lack of guilt, a lack of empathy/callousness, an unconcern about performance, and shallow or deficient affects. After a thorough examination of the ICU items, it appeared that the factor Lack of guilt and the factor Lack of empathy could be merged to form a single factor that was labeled Lack of conscience. Indeed, for some items, it was difficult to distinguish the process implicated between a lack of guilt and a lack of empathy, both being linked. Besides, these two primary constructs are part of the conscience and are often used as a definition of this latter concept (Thompson and Newton, 2010). Moreover, Frick et al. (2014b) suggested that CU traits might be associated with a deficit in the normal development of conscience, which may be a key concept of CU traits. For these theoretical models, the inclusion of a general dimension of CU traits was investigated. Therefore, we investigated two theoretical models, namely (Model 4) a second-order model with four first-order factors (Lack of empathy, Lack of guilt, Unconcern about performance, Lack of emotional expression) and a general dimension of CU traits, and (Model 5) a second-order model with three first-order factors (Lack of conscience, Unconcern about performance, Lack of emotional expression) with a general dimension of CU traits. We chose to examine second-order models rather than bifactor models, given the advantages of the second-order model regarding the interpretation of the subfactors, as well as the limitations related to the bifactor model (Watts et al., 2019). For models 4 and 5, three items of the ICU (items 12, 13, 22) were removed prior the statistical analysis. Item 12 (“Seems very cold and uncaring”) is confusing because it could refer both to a lack of emotional expression and to a lack of conscience/lack of empathic concern and is therefore impossible to place in one single factor. Also, in our opinion, item 13 (“Easily admits to being wrong”) does not fall under any of the factors; it does not refer to a lack of guilt, a lack of empathy, an unconcern about the performance or a lack of emotional expression. Waller et al. (2015) likewise reported that this item does not fit in one of the three subfactors of their factorial structure but is encompassed in the general CU traits factor in their study. Finally, item 22 (“Hides his/her feelings from others”) was deleted from our study due to the difficulty of parents to observe this process. Parents are unable to judge if their child hides his/her feelings from others, or if he/she does not feel them at all. This item seems far more appropriate for the self-reported version of the ICU at a later stage of development.

Once an adequate model was reached for the whole sample, the second aim of the study was to evaluate measurement invariance of the ICU across age period (the preschool vs. the school-aged period) and gender, in order to see if the selected factorial structure could be generalized across childhood and across gender. This investigation of measurement invariance was implemented through Multi-Group Confirmatory Factor Analysis (MGCFA). Similar to previous studies, we assumed complete invariance across gender (Essau et al., 2006; Houghton et al., 2013; Gao and Zhang, 2016; Bansal et al., 2020a; Allen et al., 2021) and partial invariance across age (Ezpeleta et al., 2013; Bansal et al., 2020a).

Finally, the third aim was to assess the external validity of the ICU scores. Accordingly, associations with measures of CU traits, externalizing behaviors, internalizing behaviors, and empathy were investigated. Regarding the model supported by the current analyses, hypotheses were different. In the case of the empirical models, it was hypothesized that the general CU score, and the Callous and Uncaring factors would show positive and similar associations with externalizing behaviors, low positive associations with anxiety and negative associations with empathy. Regarding the Unemotional scale, a different pattern of associations was expected with weak associations with externalizing behaviors, internalizing and empathy. Concerning the theoretical models, and based on the results of Koutsogiorgi et al. (2021), it was hypothesized that the Lack of empathy and the Lack of guilt factors (or the Lack of conscience factor) were associated with higher externalizing behaviors than the other subfactors. The Lack of emotional expression factor was assumed to be negatively associated with internalizing behaviors. The Unconcern about performance factor was assumed to positively correlate with externalizing behaviors, but not with internalizing behaviors nor empathy.

## Materials and Methods

### Participants

Data were collected from a sample of 437 children aged 3–9 from the French-speaking region of Belgium. Parents were recruited on social media and from Belgian schools to answer an online questionnaire. After a short description of the study and of the guarantees that the data would remain confidential, participants were requested to consent to participate. The study was approved by the Ethical Committee of Psychology of the University of Liège. The start of the online survey collected demographic information about the child, the family and the parents, and was immediately followed by validated questionnaires.

The respondents were aged 22–52 (*M* = 35.24; SD = 4.9), were principally mothers (94.3% of the sample) and 94% of the parents had, at least, completed secondary school education. This percentage is relatively high in comparison to the official indicators provided by the National Institute of Statistics in Belgium (INS), which show that approximatively 84% of the population between 25 and 54 years-old had completed a minimum level of secondary school education in 2020 (OECD, 2021). Around 84% of the respondents were living with the other parent of the child.

The average age of the 437 children for the total sample was 5.59 (SD = 1.66) and 239 were boys (54.7%). The preschool subsample included 284 children (*M*_age_ = 4.57, SD = 0.79) with 54.6% boys, while the school-aged subsample included 153 children (*M*_age_ = 7.49, SD = 01.11) with 54.9% boys. The two subsamples did not differ based on the gender of the child (χ^2^(1) = 0.004, *p* = 0.95), the gender of the informant (χ^2^(1) = 0.94, *p* = 0.33), or level of education of the informant (χ^2^(5) = 5.47, *p* = 0.36) but differed based on the age of the informant (*F* (1, 435) = 34.93, *p* < 0.001). Indeed, the parents of the preschool children were younger (*M* = 34.27, SD = 4.48) than the parents of the school-aged children (*M* = 37.07, SD = 5.14). In the total sample, 15.1% of the children had scores considered as clinical or borderline for the aggressive subscale of the *Child Behavior Checklist* (CBCL; Achenbach and Rescorla, 2000, 2001). This percentage significantly differed between the two subsamples (χ^2^(1) = 22.38, *p* < 0.001) with 9.2% of the preschool children and 26.1% of the school-aged children having clinical or borderline levels of aggressive behavior. Children with autism, developmental delay or intellectual disability were excluded from the study.

### Measures

*Inventory of Callous-Unemotional Traits* (ICU; Frick, 2004). The parent-reported preschool and school-aged versions of the Inventory of Callous-Unemotional Traits (Frick, 2004) were used in the present study, according to the age of the child. It originally consists of 24 items rated on a 4-point Likert-type scale ranging from 0 (*not at all true*) to 3 (*definitely true*). The French versions of the questionnaires were provided by the author (P. Frick). Among the questions, twelve positively worded items require reverse scoring. The preschool and the school-aged versions are identical except for item 3, which is slightly different (“seems motivated to do his/her best in structured activities” for the preschool version, “is concerned about schoolwork” for the school-aged version). Since the invariance analysis did not show a differential functioning of this item according to the versions, the two versions were considered as equivalent.

*Child Behavior Checklist* (CBCL; Achenbach and Rescorla, 2000, 2001). The preschool and the school-aged versions of the CBCL (parent-report) were used to assess internalizing and externalizing behavior. The CBCL is a questionnaire with a 3-point Likert scale (not at all, moderately, or often present), which showed high test-retest reliability, criterion and construct validity (Achenbach and Rescorla, 2000, 2001). For the current study, the anxious/depressed, the aggressive behavior and the attention problems scales were used for the analyses. Internal consistency for these three scales was, respectively, α = 0.71, 0.89, and 0.60 for the preschool sample and 0.71, 0.88, and 0.83 for the school-aged sample. Moreover, for some children (*N* = 109), 5 additional items from the preschool version of the CBCL (i.e., does not seem to feel guilty after misbehaving, punishment does not change behavior, seems unresponsive to affection, shows little affection toward people, shows too little fear of getting hurt) were used as a screening scale for CU traits. This scale demonstrated stability from ages 3–5, convergent and discriminant validity (Willoughby et al., 2011, 2014). In the present study, internal consistency was low (α = 0.50) for this scale. This CBCL CU scale was used to investigate convergent validity.

*Griffith Empathy Measure* (GEM; Dadds et al., 2008). The GEM is an established measure of affective (e.g., “seeing another child sad makes my child feel sad”) and cognitive (e.g., “my child has trouble understanding other people’s feelings”) empathy. This questionnaire, answered by a subgroup of parents from the total sample (*N* = 260) encompasses 23 items with a nine-point Likert scale. A validation study demonstrated good test-retest reliability, a stable factor structure, and convergent validity (Dadds et al., 2008). In the current study internal consistency was α = 0.68 for affective empathy (9 items) and α = 0.52 for cognitive empathy (6 items). The French version from Girard et al. (2014) was used, but this version has not yet been validated.

### Statistical Analyses

CFA were conducted using *Mplus*, version 5.21 (Muthén and Muthén, 1998). The ICU responses were considered as continuous variables and models were estimated by using the Maximum Likelihood estimator. Indeed, as described in the TALIS 2018 Technical report (OECD, 2019, p. 202) “all constructs with ordinal response categories were scaled using continuous CFA.” The data were first submitted to the three models from the current scientific literature and fit indices were compared. These three models are (Model 1) the three-factor bifactor model (e.g., Kimonis et al., 2008), (Model 2) the three-factor model (Ezpeleta et al., 2013) and finally (Model 3) the 12-item two-factor model (Hawes et al., 2014). The two alternative theoretical models were also compared with the previous ones: (Model 4) a second-order model with four latent first-order dimensions (Lack of empathy, Lack of guilt, Unconcern about performance, Lack of emotional expression) and a second-order latent factor (General CU dimension); and finally, (Model 5) a second order model with three first-order factors (Lack of conscience, Unconcern about performance, Lack of emotional expression) and a second-order latent factor (General CU dimension).

Model fit was evaluated using the χ^2^ statistic, the χ^2^/*df* ratio, the comparative fit index (CFI), the Tucker-Lewis index (TLI), the root mean square error of approximation (RMSEA) and the standardized root mean square residual (SRMR). If the *p*-value associated with the χ^2^ is greater than 0.05, the test states that the model fits the data. Due to its dependence with the sample size, the χ^2^/*df* ratio is also used, a ratio less than 2 indicating a good model fit. CFI and TLI values greater than 0.95 indicate a good model fit, whereas CFI and TLI greater than 0.90 represent a reasonable fit. Finally, RMSEA and SRMR values ≤ 0.05 can be considered as a good fit, a value of 0.08 indicates an adequate fit whereas values > 0.10 indicate an inadequate model fit (Marsh et al., 2004). As these different models cannot be considered as embedded, AIC (Akaike information criterion; Akaike, 1987), BIC (Bayesian information criteria) and the sample size adjusted BIC were used for the comparison of the models. A good fit was determined by the minimum AIC value. Internal consistency was examined via Cronbach’s alpha.

MGCFA was used to test the measurement invariance of the selected factor model of the ICU across child age (preschool vs. school-aged subsamples) and child gender. Configural invariance (whether the factor structure is the same across groups), metric invariance (whether regression coefficients are the same across groups), scalar invariance (whether intercepts are the same across groups) and strict invariance (whether the error variances are the same across groups) analyses were conducted for the first and the second-order factors, according to the procedure described by Chen et al. (2005). The authors described a seven-step process, i.e., (a) configural invariance, (b) first-order factor loadings, (c) second-order factor loadings, (d) intercepts of measured variables, (e) intercepts of first-order factors, (f) disturbances of first-order factors, and (g) residual variances of observed variables. If both metric and scalar invariance were reached, i.e., the steps (a) to (e), the mean differences on the higher order latent factor can be tested. Measurement invariance was assessed for subgroups based on gender and on age period (preschool and school-aged subsamples). Configural, metric, scalar and strict invariances were supported if fit indices of the model were acceptable and when more constrained models did not differ significantly from less constrained models. χ^2^ difference tests (i.e., likelihood ratio test) is commonly used with a cut-off criteria of Δχ^2^ *p* > 0.05 to assess measurement invariance (Svetina et al., 2020). However, given this test is highly sensitive to sample size and departures from multivariate normality, Chen (2007) suggests the following cut-off criteria: a change of ≥–0.01 in CFI in addition to a change of ≥0.015 in RMSEA as an indication of non-invariance. When invariance was not supported, modification indices were analysed to identify the non-invariant parameters. Parameters with the highest modification indices were not constrained anymore until fit indices were adequate, and when the chi-square difference test was no longer significant and/or when the differences in CFI and RMSEA were below the cut-off criteria.

To test the convergent and discriminant validity of the ICU factorial structure, latent correlations between the ICU scores and external variables (scales from the CBCL and from the GEM) were computed with Mplus in the CFA framework. To obtain the correlations between the general factor and external criteria, the second-order model was fitted. The general dimension was simply removed from this model to obtain the latent correlations with the three first-order dimensions.

## Results

### Factor Structure of the Inventory of Callous-Unemotional Traits

Table 1 summarizes the mean and standard deviation of the ICU items for the total sample and the preschool and the school-aged subsamples separately. Fit indices for the five models are presented in Table 2. The three-factor bifactor model (Model 1) presented four fit indices that reflect poor adjustment. Indeed, the χ^2^ test permitted rejection of the null hypothesis, the normed chi-square was above 2.00, the CFI and TLI indices were substantially below the threshold of 0.95, even below the more lenient threshold of 0.90. However, the RMSEA and the SRMR indicated an adequate fit. The three-factor model (Model 2) also presented a poor model fit, especially with CFI and TLI largely below 0.95 and high AIC and BIC that reflected the worst adjustment compared to the other models. Finally, the two-factor model (Model 3) presented a reasonable model fit with CFI and TLI values greater than 0.90, SRMR below 0.05, RMSEA below 0.08 and the lower AIC and BIC compared to the other models from the literature. However, the χ^2^ test permitted rejection of the null hypothesis and the normed chi-square was above 2.00. Moreover, this model, as in previous models, does not rely on a clear theoretical background. Then, it consists of only 12 items, which reduces its content validity. Finally, the two factors may be driven by a methodological variance based on item formulation rather than on a construct variance (Ray et al., 2016a). Indeed, the Callous factor largely consists of negatively worded items, while the Uncaring factor largely consists of positively worded items.

**TABLE 1:** Descriptive statistics for the full sample and for preschool- and school-aged children separately.

		Full sample (*N* = 487)		Preschool sample (*N* = 234)		School-aged sample (*N* = 153)	
				
Item content		*M*	SD	*M*	SD	*M*	SD
ICU1	Expresses his or her feelings openly (R)	2.40	0.78	0.46	0.65	0.86	0.92
ICU2	Does not seem to know “right” from “wrong”	0.59	0.87	0.67	0.90	0.44	0.80
ICU3	Is concerned about schoolwork (R)	2.46	0.72	0.45	0.66	0.69	0.80
ICU4	Does not care who he or she hurts to get what he or she wants	0.73	0.88	0.76	0.87	0.67	0.90
ICU5	Feels bad or guilty when he or she has done something wrong (R)	2.32	0.81	0.69	0.79	0.67	0.85
ICU6	Does not show emotions	0.17	0.56	0.14	0.53	0.24	0.60
ICU7	Does not care about being on time	1.27	0.98	1.38	0.99	1.05	0.93
ICU8	Is concerned about the feelings of others (R)	2.28	0.83	0.73	0.83	0.71	0.83
ICU9	Does not care if he or she is in trouble	0.76	0.92	0.81	0.92	0.69	0.92
ICU10	Does not let feelings control him or her	0.95	0.87	1.02	0.87	0.80	0.86
ICU11	Does not care about doing things well	0.56	0.80	0.54	0.81	0.58	0.77
ICU12	Seems very cold and uncaring	0.18	0.54	0.13	0.47	0.27	0.65
ICU13	Easily admits to being wrong (R)	1.63	0.88	1.65	0.88	1.61	0.89
ICU14	It is easy to tell how he or she is feeling (R)	2.26	0.84	0.61	0.78	0.97	0.91
ICU15	Always tries his or her best (R)	2.35	0.70	0.62	0.66	0.70	0.76
ICU16	Apologizes (“says he or she is sorry”) to person he or she has hurt (R)	2.13	0.84	0.84	0.79	0.93	0.94
ICU17	Tries not to hurt others’ feelings (R)	2.01	0.79	0.98	0.74	1.01	0.88
ICU18	Shows no remorse when he or she has done something wrong	0.48	0.76	0.54	0.80	0.39	0.67
ICU19	Is very expressive and emotional (R)	2.54	0.70	0.43	0.66	0.51	0.77
ICU20	Does not like to put the time into doing things well	0.78	0.85	0.67	0.79	0.98	0.93
ICU21	The feelings of others are unimportant to him or her	0.42	0.71	0.43	0.71	0.42	0.71
ICU22	Hides his or her feelings from others	0.54	0.83	0.35	0.66	0.88	0.99
ICU23	Works hard on everything (R)	2.15	0.73	0.79	0.69	0.97	0.79
ICU24	Does things to make others feel good (R)	2.26	0.75	0.70	0.73	0.79	0.78

*ICU, Inventory of Callous-Unemotional Traits; (R), reverse scored.*

**TABLE 2:** Confirmatory factor analyses: fit indices for the five models tested.

Indices	Model 1	Model 2	Model 3	Model 4	Model 5	Model 5 with MI
χ**2**, *df, p*	493.820; *df* = 187; *p* = 0.0000	780.671; *df* = 249; *p* = 0.0000	126.838; *df* = 53; *p* = 0.0000	558.482; *df* = 185; *p* = 0.0000	555.107; *df* = 186; *p* = 0.0000	227.430; *df* = 125; *p* = 0.0000
χ^2^/*df*	2.64	3.13	2.39	3.02	2.98	1.82
CFI	0.879	0.800	0.934	0.834	0.836	0.951
TLI	0.850	0.778	0.918	0.812	0.815	0.940
RMSEA	0.061 [0.055; 0.068]	0.070 [0.064; 0.075]	0.056 [0.044; 0.069]	0.068 [0.062; 0.074]	0.067 [0.061; 0.074]	0.043 [0.034; 0.052]
SRMR	0.060	0.070	0.044	0.065	0.064	0.044
AIC	20,272.749	22,611.813	10,973.459	19,929.744	19,924.369	16,730.308
BIC	20,631.783	22,917.808	11,006.998	20,203.099	20,193.644	16,991.424
SSA BIC	20,352.516	22,679.796	7164.723	19,990.476	19,984.194	16,788.321

*df, degrees of freedom; CFI, comparative fit index; TLI, Tucker-Lewis index; RMSEA, root mean square error of approximation; SRMR, standardized root mean square residual; AIC, Akaike information criterion; BIC, Bayesian information criterion; SSA BIC, sample-size adjusted Bayesian information criterion; MI, modification indices. Model 1: three-factor bifactor model, Model 2: model with three correlated factors, Model 3: two-factor model with twelve items, Model 4: second-order model with four theoretical factors, Model 5: second-order model with three theoretical factors, Model 5 with MI: second-order model with a second-order factor, three first-order factor, one methodological dimension, and three additional items deleted (6, 7, and 10).*

Fit indices of the two theoretical models (Model 4 and Model 5) were very similar and indicated poor model fit with the normed chi-square above 2.00, CFI and TLI below 0.90 and high BIC and AIC. RMSEA and SRMR values indicated adequate fit. Given the strong theoretical background underlying these two models, modification indices were scrutinized, but only for Model 5 due to several reasons. First, a model with three subfactors rather than four could be a more parsimonious solution. Additionally, items from the two factors Lack of empathy and Lack of guilt of Model 4 are sometimes difficult to distinguish between based on the process involved. Moreover, guilt and empathy are both part of the conscience, and the lack of conscience could be a central concept in the understanding of CU traits (Frick et al., 2014b), suggesting the usefulness of a Lack of conscience factor as in Model 5. Finally, proposing a 4-factor model (Model 4) might require many cross-loadings that could be easily avoided with the 3-factor model (Model 5).

Examination of Model 5 indicated two weak standardized factor loadings: (i) item 7 (λ = 0.16, *p* = 0.002) on the Unconcern about performance factor and (ii) item 10 (λ = –0.023, *p* = 0.68) on the Lack of emotional expression factor. Item 7 “doesn’t care about being on time” might be less appropriate with very young children than with school-aged children, and therefore might not work the same way in our total sample. This item was also removed in the study of Benesch et al. (2014) due to low factor loading. Also, item 10 “does not let feelings control him/her” is often problematic in other factorial structures due to poor factor loading (Ezpeleta et al., 2013; Benesch et al., 2014; Willoughby et al., 2015). This item might work differently according to the age of the child, emotional regulation typically increasing throughout development (Gullone et al., 2010). Removal of these two items did not greatly improve model fit indices (χ^2^ = 475.79, *df* = 149, *p* = 0.000; χ^2^/*df* = 3.19; CFI = 0.85; TLI = 0.83; RMSEA = 0.07; SRMR = 0.06).

**FIGURE 1:**
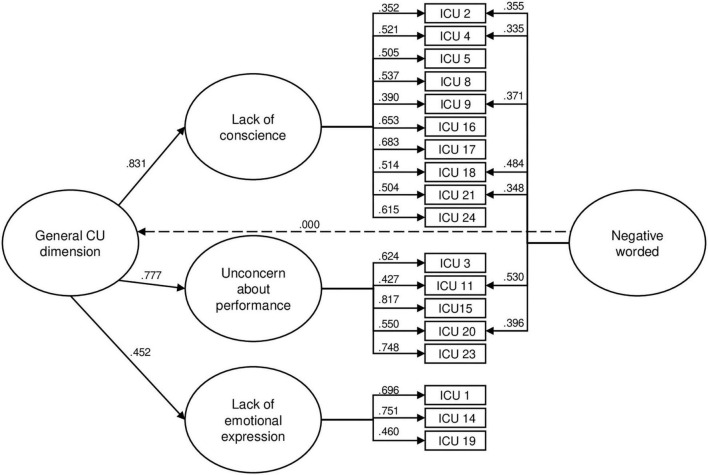
Model 5 with modifications – 18-item Second Order Model with Three First Order Factors, a Second Order Latent Factor and a Methodological Latent Factor. Items 6, 7, 10, 12, 13, and 22 were removed. Standardized factor loadings are presented.

Many residual correlations also appeared in modification indices, and many of these correlations were linked to negative worded items. This supports the hypothesis made by some authors that the Callous and Uncaring factors found in the current literature could represent a methodological artifact rather than a construct variance (Ray et al., 2016a). To overcome this issue, a methodological latent dimension encompassing these items was added to the model. Consequently, negative worded items loaded both on one of the three theoretical factors and on the methodological dimension, while positive items only loaded on one of the three factors. Initially, item 17 (“Tries not to hurt other’s feelings”) was included in this dimension. However, as it did not load significantly on the methodological dimension, it was removed. Indeed, after further examination, a French linguistic subtlety regarding the negation in the wording of this item made it different from the other negative worded items. It should be noted that the methodological dimension is by definition orthogonal to the second-order dimension. Item 6 (“Does not show emotions”) was also deleted due to a cross-loading (Lack of emotional expression and Lack of conscience) as parents may indeed interpret this item in two different ways. On the one hand, the child is able to feel emotions but does not express them for different reasons, such as an avoidant attachment profile (Cooke et al., 2019). In this case, item 6 corresponds to a lack of emotional expression. On the other hand, the child does not show emotions because he/she does not feel them, and therefore neither feels empathy and guilt, which corresponds to a lack of conscience. The final model with 18 items, three factors of first order, one second-order dimension and one methodological dimension (Figure 1) demonstrated a good fit (χ^2^ = 227.430, *df* = 125, *p* = 0.00; χ^2^/*df* = 1.82; CFI = 0.95; TLI = 0.94; RMSEA = 0.043; SRMR = 0.044). For this model, the ICU general dimension demonstrated adequate internal consistency (α = 0.85). Cronbach’s alphas for subfactors were adequate for Lack of conscience (α = 0.81) and Unconcern about performance (α = 0.78), and marginal for the Lack of emotional expression factor (α = 0.67), probably due to the small number of items included in this factor.

### Measurement Invariance

MGCFA with preschool (3–5 years old; *M* age = 4.57, SD = 0.79, *N* = 284) and school-aged (6–9 years old; *M* age = 7.49, SD = 1.11, *N* = 153) subsamples was then conducted based on Model 5 after modifications. Results from measurement invariance analysis across age are presented in Table 3. The fit indices for the configural model and the metric models (i.e., steps b and c) were adequate. Indeed, even if the chi-square difference test was significant for step c (second-factor loadings invariance), the changes in CFI and RMSEA were inferior to the cut-off criteria suggested by Chen (2007). Only partial scalar invariance was reached, as the intercept of the Lack of emotional expression first-order factor required to be separately estimated for the two subsamples. Model fit was subsequently acceptable and the difference in the model fit was in accordance with the cut-off criteria. While the intercept of the Lack of emotional expression factor is settled to 0 by default for the preschool subsample, the non-standardized intercept estimate was 0.28 (SE = 0.07, *p* < 0.001) in the school-aged sample. Given the fact that partial scalar invariance was reached, comparisons of means for the second-order factor (CU general dimension) and the methodological dimension were allowed between the two subsamples. However, strict invariance was not reached, even in freeing the residual variances of items 1 and 20. As the means of the second-order factor (the general dimension) and the methodological dimension were fixed to 0 by default for the preschool sample, the means for the school-aged sample were 0.143 (*p* = 0.208) for the general dimension and –0.0273 (*p* = 0.04) for the methodological dimension. Thus, the mean of the general dimension did not differ across age, but school-aged children had significantly lower scores at negative-worded items than preschool children.

**TABLE 3:** Multiple group measurement invariance analysis across the age period.

	Model	Release of constraints	*χ^2^*	*df*	CFI	TLI	RMSEA	SRMR	Δχ^2^	Δ*df*	*p*	ΔCFI	ΔRMSEA
a.	Configural, no constraints	–	352.21	249	0.951	0.940	0.044	0.053					
b.	First-order factor loadings	–	378.94	270	0.948	0.942	0.043	0.032	26.72	21	0.18	–0.003	–0.001
c.	Second-order factor loadings	–	390.09	272	0.941	0.937	0.045	0.034	11.15	2	0.00	–0.007	0.002
d.	Intercepts of measured variables	–	425.43	286	0.934	0.929	0.047	0.066	35.34	14	0.00	–0.007	0.002
e.	Intercepts of first-order factors	–	456.40	288	0.920	0.915	0.052	0.070	30.98	2	0.00	–0.014	0.005
		D3	437.51	287	0.929	0.924	0.049	0.068	12.08	1	0.00	–0.005	0.002
f.	Disturbances of first-order factors	–	438.57	290	0.930	0.926	0.048	0.069	1.06	3	0.79	0.001	–0.001
g.	Residual variances of observed variables	–	523.34	308	0.898	0.899	0.057	0.075	84.77	18	0.00	–0.032	0.009
		ICU1 ICU20	479.10	306	0.918	0.918	0.051	0.072	40.53	16	0.00	–0.012	0.003

*df, degrees of freedom; CFI, comparative fit index; TLI, Tucker-Lewis index; RMSEA, root mean square error of approximation; SRMR, standardized root mean-square residual; Δ, difference in model fits between different stages of invariance; D3, Lack of emotional expression (first-order factor).*

Measurement invariance of the selected model across gender was also evaluated, and the results are presented in Table 4. The configural invariance model provided a good fit to the data in terms of all the indices, as well as the metric invariance and scalar invariance models. Given the fact that scalar invariance was reached, comparisons of means for the second-order factor (CU general dimension) and methodological dimension were allowed between the two subsamples. Strict invariance was not reached, even when freeing the residual variances of item 3. Indeed, the chi-square difference test was significant, and the CFI change exceeded –0.01. As the means of the second-order factor (the general dimension) and the methodological dimension were set to 0 by default for boys, the means for the girls subsample were, respectively, equal to –0.134 (*p* = 0.003) for the general dimension and to 0.04 (*p* = 0.38) for the methodological dimension. Thus, the mean of the general dimension differed across gender, with boys having higher scores on the ICU than girls, similar to previous studies.

**TABLE 4:** Multiple group measurement invariance analysis across gender of the child.

	Model	Release of constraints	χ2	*df*	CFI	TLI	RMSEA	SRMR	Δχ^2^	Δ*df*	*p*	ΔCFI	ΔRMSEA
a.	Configural, no constraints	–	372.25	248	0.941	0.927	0.048	0.055					
b.	First-order factor loadings	–	394.15	269	0.940	0.930	0.046	0.059	21.90	21	0.41	–0.001	–0.002
c.	Second-order factor loadings	–	394.32	271	0.941	0.933	0.046	0.059	0.17	2	0.92	0.001	0.000
d.	Intercepts of measured variables	–	412.65	285	0.939	0.934	0.045	0.060	18.33	14	0.19	–0.002	–0.001
e.	Intercepts of first-order factors	–	413.79	287	0.939	0.935	0.045	0.060	1.14	2	0.56	0.000	0.000
f.	Disturbances of first-order factors	–	424.30	291	0.936	0.933	0.046	0.063	10.51	4	0.03	–0.003	0.001
g.	Residual variances of observed variables	–	484.48	310	0.917	0.918	0.051	0.072	60.17	19	0.00	–0.019	0.005
		ICU3	468.00	309	0.924	0.925	0.049	0.070	43.70	18	0.00	–0.012	0.003

*df, degrees of freedom; CFI, comparative fit index; TLI, Tucker-Lewis index; RMSEA, root mean square error of approximation; SRMR, standardized root mean-square residual; Δ, difference in model fits between different stages of invariance.*

### External Validity

Table 5 shows latent correlations between the scores of the second-order model of ICU and external measures. Correlations were presented by subsample (preschool and school-aged subsamples), in order to investigate the differences during the child development. Moreover, invariance analyses showed that the intercept of the Lack of emotional expression factor differed between the two groups. Finally, items from the CBCL scales vary between the preschool and the school-aged versions and therefore are not comparable.

**TABLE 5:** Latent correlations between the ICU second-order and first-order factors and external variables according to the age period.

	Preschool period					School period				
		
	General	Method.	Conscience	Performance	Emotional expression	General	Method.	Conscience	Performance	Emotional expression
Agg. behavior	0.62***	0.21***	0.59***	0.44***	0.09	0.63***	0.38***	0.61***	0.42***	0.41***
Anxious/depressed	0.29***	0.08	0.22**	0.19**	0.29***	0.26**	0.26*	0.12	0.19*	0.31***
Attention problems	0.43***	0.41***	0.29***	0.41***	0.06	0.55***	0.59***	0.39***	0.58***	0.22*
CU scale	0.75***	0.38***	0.74***	0.50***	0.22*	–	–	–	–	–
Affective empathy	–0.28*	–0.25	–0.31**	–0.13	–0.21	–0.35***	–0.12	–0.39***	–0.13	–0.20*
Cognitive empathy	–0.30*	–0.27	–0.39**	–0.10	–0.01	–0.64***	–0.11	–0.62***	–0.41***	–0.46***

*General, second-order factor of the ICU corresponding to a general dimension of CU traits; Method., methodological dimension encompassing the negative-worded items; Conscience, lack of Conscience; Performance, unconcern about performance; Emotional expression, lack of Emotional expression; Agg. behavior, aggressive behavior.*

*p ≤ 0.05, **p ≤ 0.01,***p ≤ 0.001.

As expected, the general dimension significantly and positively correlated with all the scales of the CBCL (aggressive behavior, anxious/depressed, attention problems) in a similar way for the two subsamples. Specifically, the strongest association is observed with aggressive behavior in the two subsamples (*r* = 0.62 and.63, *p* < 0.001) and the associations with the anxious/depressed scales are small but positive. The general dimension is also strongly and positively correlated to the CBCL CU scale in the preschool sample (*r* = 0.75, *p* < 0.001), supporting the fact that they measure a similar construct. Finally, the general dimension of CU traits is significantly and negatively associated with affective and cognitive empathy in the two subsamples, even if the association with cognitive empathy is much stronger in the school-aged subsample (*r* = –0.62, *p* < 0.001). In this subgroup the general dimension is more closely associated with cognitive empathy than with affective empathy (*r* = –0.35, *p* < 0.001).

Regarding the first-order factors, the Lack of conscience factor showed a similar pattern as the general dimension of CU traits with the strongest associations (in comparison to the other first-order factors) with the aggressive behaviors, affective and cognitive empathy in the two subsamples and with the CU CBCL scale in the preschool subsample. A moderate association was also found with the attention problems scale, regardles sof the child’s age. Finally, the Lack of conscience factor was significantly associated to higher scores on the anxious/depressed scale only in the preschool-aged subsample (*r* = 0.22, *p* < 0.001). The Unconcern about performance factor was most strongly associated with attention problems in the two subgroups, in comparison to other first-order factors, with moderate associations (*r* = 0.41–0.58, *p* < 0.001). This factor was also positively associated with the aggressive behavior (*r* = 0.44–0.42, *p* < 0.001) and the anxious/depressed scales in the two subgroups, and with the CU CBCL scale in preschoolers. However, it was not associated with empathy measures, except with cognitive empathy only in the school-aged subsample (*r* = –0.41, *p* < 0.001). The Lack of emotional expression factor presented a different pattern of associations between the two subgroups. Low to moderate significative correlations were found with aggressive behavior (*r* = 0.41, *p* < 0.001), attention problems (*r* = 0.22, *p* = 0.03), affective empathy (*r* = –0.20, *p* = 0.05) and cognitive empathy (*r* = –0.46, *p* < 0.001) but only in the school-aged subsample. However, this factor positively correlated with the anxious/depressed scale regardless of the child’s age (*r* = 0.29–0.31, *p* < 0.001).

## Discussion

This study aimed at examining the factorial structure, the measurement invariance and the validity of the French parent-reported version of the ICU in a sample of Belgian preschool and school-aged children. Models that were based on both empirical evidence and theoretical framework were investigated, and item wording was considered. This study contributes to the current literature in: (1) supporting a theoretical factorial structure in a mixed sample of children aged 3–9, with good fit indices; (2) showing measurement invariance across gender and, to a lesser extent, across development of the child; (3) supporting the relevancy of considering a general dimension of CU traits as well as item wording; (4) finding evidence for the validity of the French parent-reported version of the ICU.

The factorial structure of the ICU is still under debate in the current literature. The two most supported structures are a three factor-bifactor model (Essau et al., 2006) and a two-factor model (Hawes et al., 2014). In the current study, the bifactor model presented poor model fit, contrary to the two-factor model which showed the best fit compared to the other empirical factorial models. This is consistent with previous studies on preschool and school-aged children (Hawes et al., 2014; Kimonis et al., 2016; Bansal et al., 2020b). However, the deletion of half of the items in comparison to the original ICU increased model fit but decreased content validity, and the model did not present a satisfactory fit with all the fit indices investigated. Furthermore, several authors have questioned the possibility that the two factors are simply the product of a methodological artifact, given that one factor primarily encompasses negatively worded items while the second factor primarily encompasses positively worded items (Ray et al., 2016a; Cardinale and Marsh, 2020). This factorial structure, as most factorial structures found in the literature, does not rely on a strong theoretical framework but is mainly driven by the data. For example, some items which evaluate the same process such as a lack of empathy loaded on different factors (Paiva-Salisbury et al., 2017). Finally, this model does not consider the ICU as a unidimensional measure. Therefore, two theoretical models, respectively, with three and four factors, were investigated. They were based on the LPE specifier criteria from the DSM-V to form factors that make sense at both a theoretical and clinical level. Indeed, these criteria were based on the CU subscale of the APSD (Frick and Hare, 2001) which also served to construct the ICU. The results showed that an 18-item second order model with three first-order factors (Lack of conscience, which encompasses the criteria Lack of guilt and Lack of empathy from the LPE specifier; Unconcern about performance; Lack of emotional expression), a second-order latent factor (representing a general CU dimension) and finally a methodological latent factor (encompassing the negative worded items) presented the best fit in this study. This factorial structure is consistent with the one found via exploratory factor analysis by Benesch et al. (2014) in a clinical sample of school-aged boys, although this latter did not encompass a general dimension of CU traits (3 similar subfactors: Callousness/Lack of guilt or remorse; Unconcern about performance; Unemotional). Other studies have investigated a factorial structure based on the LPE specifier criteria. A number of them have failed to confirm this model with adolescent offenders (Kimonis et al., 2008) or middle-aged children (Bansal et al., 2020a). Indeed, the model proposed by Kimonis et al. (2008) presented an inadequate model fit, which is in line with our results. Furthermore, estimation problems due to an overly high correlation between factors were reported by Bansal et al. (2020a). However, two other studies that took into account the item wording succeeded in having an accurate fit for their model with four correlated factors (Kliem et al., 2020; Koutsogiorgi et al., 2021), but only with the self-reported version of the ICU. Again, the correlations between the factors Lack of empathy and Lack of guilt were very strong in these studies (*r* = 0.88–0.93). The items from these two factors might be too closely related to the same psychological processes, and therefore would be better represented by a single factor grouping them together (named Lack of conscience in the present study). Indeed, they are both moral emotions as part of the conscience (Thompson and Newton, 2010) and could be a core concept for the understanding of the development of CU traits (Frick et al., 2014b). Moreover, problems in guilt and empathy have been shown to be the best indicators of CU traits (Frick, 2009). Consistent with this hypothesis, the 12-item 2-factor structure of Hawes et al. (2014) showed that after IRT analysis, most of the remaining items were relative to a lack of guilt or empathy.

The current study is the first to empirically support the relevance of considering the item wording in the factorial structure of the parent-version of the ICU. Indeed, the creation of a methodological dimension encompassing the negative worded items resulted in a considerable improvement in the fit indices of our model. These results are similar to previous results with the self-reported version of the ICU (Paiva-Salisbury et al., 2017; Kliem et al., 2020; Koutsogiorgi et al., 2021). This methodological dimension controls the fact that positive and negative worded items differ in item response patterns, such that negative worded items (indicating low levels of prosocial emotions) are less difficult to endorse, and thus are more discriminating at lower levels of CU traits than positive items (indicating high levels of callousness). Positive items are therefore more discriminating at higher levels of CU traits (Ray et al., 2016a). This methodological dimension could help future research to clarify the factorial structure of the ICU without biases linked to the item formulation.

While a large majority of previous studies failed to confirm a theoretical model (with three or four factors based on the LPE specifier) with a general dimension of CU traits (Kimonis et al., 2008; Kliem et al., 2020; Koutsogiorgi et al., 2021), our study provided support for a second-order factor representing a general dimension of CU traits. This added evidence for the unidimensionality of the concept of CU traits as based on the LPE specifier criteria, even if a common factor extracted from these constructs does not necessarily mean that this common factor exists in reality (Bonifay et al., 2017). A general dimension of CU traits could be useful in research and clinical contexts. Indeed, several authors have reported the latent general CU score to show the best predictive and concurrent validity, in contrast to subfactors which have shown inconsistent correlations with external variables (Waller et al., 2015; Ray and Frick, 2020; Thøgersen et al., 2020). Moreover, this general dimension seems crucial since it demonstrated the ability of the ICU to diagnose the global phenomenon of CU traits as defined by the LPE specifier, and not only distinct constructs (such as Callous and Uncaring in the 2-factor structures). Modeling a general dimension of CU traits should consequently be a concern for future studies investigating the factor structure of the ICU. In the literature several studies have failed to report a general dimension of CU traits (as in the 2-factor model, for example) but have still calculated a summed total score in order to perform other analyses. This inconsistency should be avoided, as secondary analyses of the latent variables should be consistent with the underlying psychometric model.

In this study, we chose to investigate second-order models rather than bifactorial models, which are often tested in the literature about the ICU. Indeed, the bifactorial models are characterized by a tendency to overfit data and produce out-of-range parameter estimates, in addition to the difficulty of interpreting subfactors from a clinical point of view (Bonifay et al., 2017; Watts et al., 2019). Nevertheless, it is not our intention, or recommendation, to reject the bifactor model as its use might be more adequate for the investigation of the effects of the specific subdimensions rather than the effects of the general dimension (Chen et al., 2006). Future research should test the current factorial structure within a bifactorial model to investigate the differences between both approaches.

Measurement invariance results demonstrated that this structure was invariant across boys and girls, and to a lesser extent across child age. Specifically, the structure of CU traits did not differ between boys and girls, even though boys had higher scores on the general dimension than girls. This is consistent with past work with the self-reported version (Essau et al., 2006; Ciucci et al., 2014; Pihet et al., 2015) and parent-reported version of the ICU (Hawes et al., 2014; Gao and Zhang, 2016; Bansal et al., 2020b), no matter the factorial structure investigated. Regarding child age, partial invariance was found, with one parameter (intercept of Lack of emotional expression factor) needed to be separately estimated in the preschool and school-aged subsamples in order to achieve an adequate fit. These results are consistent with past work which also showed partial measurement invariance across age in preschoolers (Ezpeleta et al., 2013) and middle-aged children (Bansal et al., 2020a), although this is the first study to our knowledge to specifically investigate measurement invariance across preschool and school-aged children. However, the non-invariant parameters differ between studies, which supports the need for further investigation of measurement invariance across age, especially up to the end of childhood. Indeed, our results might imply that the factorial structure of the ICU could change slightly during development, particularly for the Lack of emotional expression. The means for the general dimension did not differ between the preschool and the school-aged children in the current study. This is consistent with the results of Bansal et al. (2020a) and Ezpeleta et al. (2013), which showed that the means of their two or three factors did not differ between age. However, they did not include a general dimension of CU traits. Our results support the use of our factorial structure in early and middle childhood.

In terms of external validity, the general dimension of ICU showed the expected correlations with externalizing behaviors (aggressive behavior and attention problems) and with affective and cognitive empathy regardless of the age of the child. The general dimension is also positively associated with negative affects (such as anxiety and depression). These results are consistent with the meta-analysis of Cardinale and Marsh (2020). A strong correlation was also found with the CU subscale of CBCL in the preschool-aged subsample, adding evidence that they measure the same construct. Consistent with Benesch et al. (2014), the subfactor Lack of conscience was the most correlated with aggressive behavior and empathy variables in comparison to the other subfactors. However, the Unconcern about performance factor was the most associated with attention problems, which is also consistent with previous studies (Benesch et al., 2014; Koutsogiorgi et al., 2021). This factor was moderately associated to aggressive behavior, which is similar to the results of Koutsogiorgi et al. (2021) but in contradiction with Benesch et al. (2014) who found non-significant association between these two variables. Finally, low positive correlation was found with internalizing behaviors, while Benesch et al. (2014) found an inverse correlation. These results should be carefully explored in future studies. The Lack of emotional expression factor showed a very different pattern of associations in comparison to the other subfactors, but only in the preschool-sample. Indeed, it only positively correlated with internalizing behavior and with the CBCL CU scale. These results are consistent with the majority of studies investigating this subscale, reporting low internal consistency and inconsistent associations with external criteria variables (e.g., Essau et al., 2006; Benesch et al., 2014; Kimonis et al., 2016; Ezpeleta et al., 2017; Cardinale and Marsh, 2020). The Unemotional subscale seems to function differently in comparison to the others subscales, and might evaluate different constructs. Consequently, the two-factor models have often deleted this factor, or only kept one item from it (Hawes et al., 2014; Waller et al., 2015). Likewise, in this study we only kept 3 items from this factor, because other items loaded on several factors. Moreover, the factor loadings indicated that this factor is less explained by the general dimension in comparison to the other two first-order factors in this study.

Several hypotheses can be made to explain the poor performance of this scale. First, items that were kept in this model refer to a lack of emotional expression, and not to a lack of emotional activation, this latter characterizing CU traits (for a review, see Frick et al., 2014b). Parents could therefore misunderstand these items as indicating a shy or withdrawal attitude (Cardinale and Marsh, 2020). Moreover, the differentiation between a lack of emotional expression and a lack of emotional activation in the child could be complicated for the parents, as this latter refers to internal processes but could result in observable behavior of low emotional expression. For example, children that present an avoidant attachment profile consequently minimize their expression of emotions (Cooke et al., 2019) even though they present a significant physiological and emotional activation (Borelli et al., 2014). On the contrary, a child with high levels of CU traits could present a lack of emotional expression but associated with a low physiological and emotional activation. Thus, items from the ICU might not be adequate in detecting the shallow or deficient affects criterion from the DSM-V LPE specifier. Second, it could be questioned if this LPE specifier criterion, and therefore the ICU items of this factor, is adequate itself in describing children with CU traits. Indeed, children with CU traits might not be “cold” of void of emotional expression in all contexts and for all forms of emotional experiences (Northam and Dadds, 2020). Items of the ICU might fail to capture the specificity of the emotional/affective deficits of children with CU traits. Third, inconsistencies about the Lack of emotional expression/unemotional factor found in the literature and in this study could also result from the heterogeneity within the group of children with high levels of CU traits. Indeed, two variants were highlighted, based on the absence (primary variant) or the presence (secondary variant). Authors suggest that while the primary variant could experience hypo-arousal to negative affects and so limited emotional response to the distress of others, the secondary variant could be characterized by hyperarousal and sensitivity to negative affects and so by negative emotionality and impulsivity (Kimonis et al., 2012; Ezpeleta et al., 2017). According to this theory, only the primary variant might present deficits in emotional responsiveness and consequently high scores at the Lack of emotional expression factor, while the secondary variant might obtain low scores on this factor. This hypothesis is supported by Kimonis et al. (2013) who found higher scores on the Unemotional factor for the primary variant than for the secondary variant in a sample of incarcerated boys. However, the distinction between the two variants based on this factor could be less obvious in young child samples when compared to adolescent samples. Indeed, emotional responsiveness, expression and regulation evolve throughout child development (Labouvie-Vief et al., 2007; Stegge and Terwogt, 2007; Gullone et al., 2010; Pollak et al., 2019) and emotional deficits seem to be less salient in children than in adolescents with CU traits (Northam and Dadds, 2020). This might explain why the pattern of correlations between the Lack of emotional expression and external variables was so different between preschool and school-aged children, and why the intercept of this factor had to be separately estimated for both subsamples in this study. Other measures to assess emotional activation and responsiveness could be used to further investigate this area and items of the Lack of emotional expression factor could be reworded by taking into account various contexts and emotional experiences, and by relying on specific and observable behaviors (Kimonis et al., 2015).

The present results supported the usefulness of the DSM-V LPE specifier in the context of the evaluation of CU traits in childhood. Indeed, the different criteria from this specifier were found using the ICU in the form of 3 factors participating in a general dimension of CU traits. This consistency between the diagnostic criteria and the assessment instrument (the ICU) is essential for clinicians and researchers working with children exhibiting CU traits as it enhances the ability to translate ICU scores from research to practice. Indeed, our study supports the fact that the ICU would measure a CU trait construct that is unidimensional. This is in line with current literature showing the utility of CU traits in delineating a subgroup particularly at risk of antisocial outcomes and psychopathy (Lynam et al., 2007). This general dimension of ICU could help clinicians to improve the diagnosis of CU traits based on the LPE specifier criteria. Moreover, the clinicians could use the subfactor scores to establish distinct profiles within the group of children with CU traits, given the results showing that the subfactor displays distinct associations with external correlates. Therefore, in addition to helping with diagnosis, this structure could also guide the subsequent intervention. However, questions could be raised about the LPE criteria and specifically which of them are really at the core of CU traits, and the best way of distinguishing this phenomenon from others. Given that 2 out of 4 factors are required for the LPE specifier, it could raise doubts about whether the factors Unconcern about performance and Lack of emotional expression/Unemotional would be sufficient to distinguish a subgroup of children with CU traits. Indeed, while the former factor is little studied in current literature about CU traits, the latter factor could vary according to the age of the child, the context, the primary/secondary variant, and is not easy to assess (at least in the case of other-reported questionnaires). On the contrary, children may necessarily require lack of guilt and empathy to present CU traits. This should be the subject of further research to determine which characteristics are really central and essential to the definition of CU traits, how best to assess them and if the DSM-V LPE specifier needs to be refined, especially since children may exhibit CU traits without necessarily having a diagnosis of Conduct Disorder (Fontaine et al., 2011; Kumsta et al., 2012; Ezpeleta et al., 2013; Frick et al., 2014a).

## Limitations

These results must be considered within the context of several study limitations. First, external validation was based solely on questionnaire data. Further research is needed to investigate associations with physiological and neurobiological measures (e.g., amygdala activation) and laboratory tasks (e.g., evaluation of empathy or emotion recognition). Moreover, the same parent answered all the questionnaires for external variables and the ICU. Therefore, the correlations could be inflated by shared method variance. Some factors could also limit the generalization of these results. Our sample comes from the Belgian general population but presents a higher level of education than the mean, and therefore may not be representative of the entire population. This is certainly due to the recruitment procedure via social networks. Moreover, even though our community sample included children with clinical levels of externalizing behaviors, it is not known how these results could be generalized in clinical samples, in samples from different countries, or in children with more sociodemographic risk factors. Besides, only parent ratings were collected, so results cannot be generalized to the other versions of the ICU (teacher-report or self-report). Additionally, most of the respondents were the children’s mothers, so we were unable to examine measurement invariance across parent gender. However, Bansal et al. (2020a) reported partial invariance across parent sex in a community sample of children aged 5–12. Further research should investigate this question and include different informants in order to establish additional information about the factorial structure of the ICU. Finally, they were several external scales that demonstrated less than ideal internal consistency (i.e., Cronbach’s α below 0.80), although similar internal consistencies were found in previous studies (Ezpeleta et al., 2013; Kimonis et al., 2016). Therefore, these findings need to be further scrutinized in future investigations.

## Conclusion

In conclusion, the current study promoted an 18-item second-order model of ICU based on the DSM-V specifier “with limited prosocial emotions” in a Belgian sample of preschool and school-aged children. This factor model, consisting of three factors of first order (Lack of conscience, Unconcern about performance, Lack of emotional expression), one second order dimension (General dimension of CU traits) and one methodological dimension encompassing negative worded items, exhibited good fit indices. This model also demonstrated support for measurement invariance across gender and age and external validity. Thus, our study supports the use of the French-version of the parent-reported version of the ICU in early and middle childhood. Future research should continue to evaluate the parent-reported version of the ICU while taking into account the theoretical and clinical characteristics of CU traits, and therefore constructing consistent models. The consideration of item wording seems essential to clarify the structure of ICU, as well as the possibility of including a general dimension within a second-order or a bifactorial model. Moreover, additional work is needed to investigate both the specific correlates of subfactors and the general dimension of CU traits with external variables. Finally, future research should focus on the appropriateness of the DSM-V LPE specifier criteria for detecting children with high levels of CU traits, and adapt the ICU items accordingly.

## Data Availability Statement

The raw data supporting the conclusions of this article will be made available by the authors, without undue reservation.

## Ethics Statement

The studies involving human participants were reviewed and approved by Ethical Committee of Psychology of Liège University. The patients/participants provided their written informed consent to participate in this study.

## Author Contributions

MP and MS contributed to the study conception and design. CM and MP performed the material preparation and analyses. MP drafted the initial version of the manuscript and performed the data collection. All authors commented on previous versions of the manuscript, read and approved the final manuscript.

## Conflict of Interest

The authors declare that the research was conducted in the absence of any commercial or financial relationships that could be construed as a potential conflict of interest.

## Publisher’s Note

All claims expressed in this article are solely those of the authors and do not necessarily represent those of their affiliated organizations, or those of the publisher, the editors and the reviewers. Any product that may be evaluated in this article, or claim that may be made by its manufacturer, is not guaranteed or endorsed by the publisher.

## Footnotes

1 Measurement invariance indicates that the same underlying construct is being measured across groups or across time and is a requirement before means comparisons (Cheung and Rensvold, 2002).
